# Case report: Diagnosis and treatment of a chinchilla’s old radius and ulna fracture

**DOI:** 10.1186/s44149-023-00072-0

**Published:** 2023-03-23

**Authors:** Siyu Long, YuJing Qiao, Hua Cao, Tiantian Qiu, Yuji Chen, Yaoqin Shen

**Affiliations:** 1 College of Veterinary Medicine, Huazhong Agricultural University, Wuhan, 430070 People’s Republic of China; 2 Veterinary teaching hospital of Huazhong Agricultural University, Wuhan, China

**Keywords:** Chinchilla, Fracture, Radius and ulna, Internal fixation

## Abstract

The cortex of the limb bones of chinchillas is very thin and brittle, so it is prone to fractures of the limb bones, among which fractures of the tibia, radius and ulna are the most frequent types. When a chinchilla has a closed fracture, it can be immobilized with a splint, cast, or bandage. If the broken end of the fracture pierces the skin, it is best to choose internal fixation or external fixation brackets for treatment. In this report, a 0.661 kg, 2-year-old male uncastrated chinchilla was presented to the Veterinary Teaching Hospital of Huazhong Agricultural University due to an old fracture of the right forearm. With the consent of the owner, we decided to use a 25-gauge needle as an IM pin to fix the fracture. Ten days after surgery, the wound had healed well, and the limb could support body weight, but the palm did not show a grasping position. Twenty four days after the operation, the affected limb had not regained the ability to grasp. The X-ray showed a slight rotation of the IM pin and good callus growth in the ulna, but not in the radius. One month after the operation, it was found that the function of the affected limb of the chinchilla was normal and the grasping ability was restored through follow-up consultation and the return visit.

## Background

Chinchillas are becoming increasingly popular as companion animals due to their long life span, hardiness and simple husbandry requirements (Hsu et al. 2015). As a result, common diseases in chinchillas have also been gaining attention in recent years (Martel et al. 2022; Mans and Donnelly 2013; DeCubellis and Graham 2013). Among them, the cortex of the limb bones of chinchilla is very thin and brittle (Mitchell and Tully 2008), so it is easy to fracture the limb bones (Krautwald-Junghanns et al. 2011), such as limbs stuck in wire fence cages, improper restraint, etc. The hindlimbs are more frequently fractured than the forelimbs, which may be related to the chinchilla’s half- bounding locomotion pattern (Desprez et al. 2016). When chinchillas exercising, the hind limbs are used to jump at the same time, and then the front limbs are used to land on the ground (Lammers and German 2002). When a fracture occurs, the appropriate treatment method should be selected according to the situation of the fracture. When the skin is intact, external fixation with a splint, cast or bandage may be considered (Miwa and Calvo Carrasco 2019). Because there are few muscles covered by the limbs of chinchillas, the fractured ends can easily pierce the skin and form open wounds (Mitchell and Tully 2008). Therefore, in the case of skin damage, internal fixation and installation of external fixation brackets are reasonable fixation methods. External fixation is also a commonly used method of fracture fixation, which can provide adequate stability to the affected limb (Sánchez-Migallón Guzmán and Kapatkin 2021). The diameter of the limbs of chinchillas is very limited, and the internal fixation needs to select a small enough IM pin for fixation to avoid further rupture of the cortical bone. The forelimbs of chinchillas are slenderer than the hind legs and have thinner skin and muscle layers, so using IM pin and wire cerclage is not the first choice. When the radius and ulna are fractured and there is no skin injury, external fixation can often provide sufficient stability, but chinchillas will chew on the external fixation material, resulting in insufficient stability and ultimately failure of the treatment. In this case, because chinchillas were diagnosed with an old fracture, surgery was first required to destroy the formed callus. Therefore, internal fixation is the only option. In the absence of suitable internal fixation materials, we used 25-gauge needles instead of IM pin for intramedullary fixation.

This case report describes the surgical management and outcomes of an old fracture of the radius and ulna in a chinchilla. This is also the first report of using a syringe needle to treat old fractures of the radius and ulna in chinchillas. It is hoped that this case report can provide a reference for the diagnosis and treatment of chinchilla fractures.

## Case presentation

### Case history

A 0.661 kg, 2-year-old male, uncastrated chinchilla presented with a fractured right forearm. According to the owner’s description, the fracture occurred 2 months ago. The chinchilla lived in a cage made of iron wire. Due to being frightened, the right forelimb got stuck in the gap of the cage and fractured. By the time of consultation, the right forelimb could not support weight and maintain balance, and chinchillas often fell down and screamed during the process of climbing and jumping. Chinchillas were fed timothy grass and a commercial diet daily. Its appetite and mental state were normal until presentation.

### Clinical findings

**Fig. 1:**
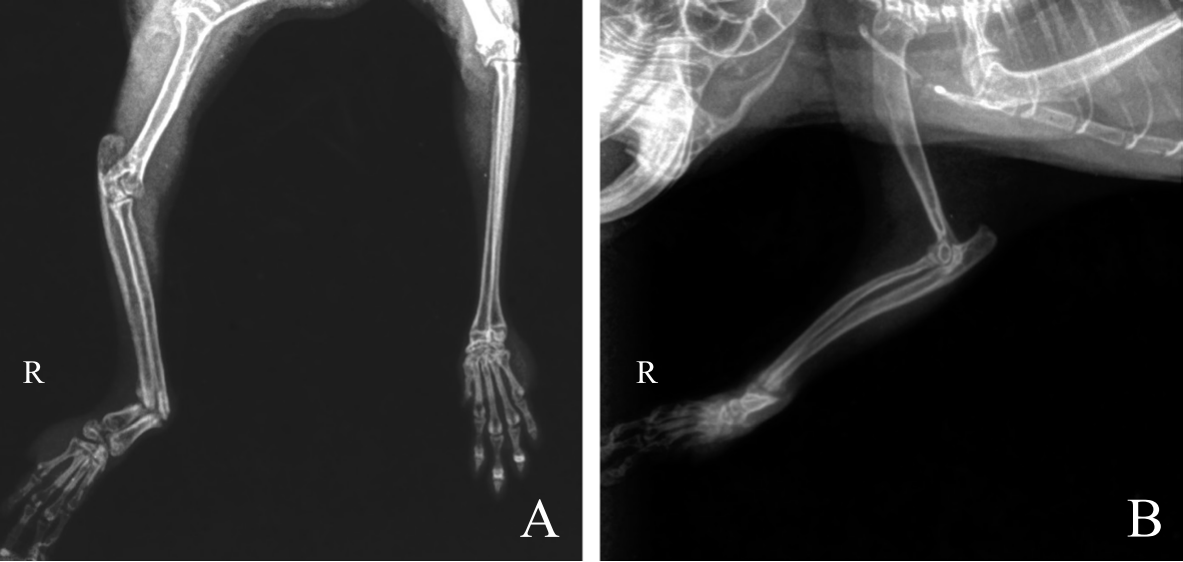
X-ray results of the affected forlimb. **A** Dorsoventral view; **B** Right lateral view. A and B showed oblique fractures of the radius and distal ulna with a distinct callus

Structural abnormalities were observed near the wrist joint of the right forelimb of the chinchilla, and the right forelimb didn’t support body weight when lean forward. The palm was obviously curved to the right posterior side, and the broken end was thickened and inactive. Motor and neurological examination of the right forelimb demonstrated a normal amputation pain response, normal stretching, and ability to grasp food. Based on the past medical history and general physical examination, the initial diagnosis was a fracture of the radius and ulna of the right forelimb with malunion. X-rays were recommended for further examination to confirm the diagnosis. Two radiographs were taken, using the dorsoventral and right lateral views, respectively, and the diagnosis was finally made of a distal oblique fracture of the radius and ulna (Fig. 1A, B). There are obvious callus images around the broken end. The diameter of the radial medullary cavity was measured to be 0.7 mm.

### Treatment

**Fig. 2:**
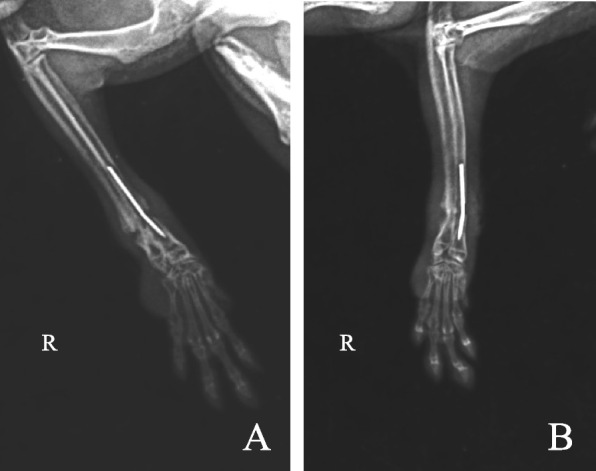
Postoperative X-ray results. **A** Taken immediately after surgery; **B** Taken one month after surgery. A showed good reduction of the radius and ulna. B showed that the needle has rotation and callus growth of the radius and ulna. The ulna aligns well, but the ulna has not fully healed

Before surgery, chinchilla was pre-treated with enrofloxacin (10 mg/kg, SC, BAYTRIL, BAYER, Germany), 6 mL normal saline (20 mL/kg, SC), heated to 37°C, and injected subcutaneously at multiple points. Anesthesia was delivered through a respiratory mask with 5% isoflurane (R 620, RWD, China), while oxygen was given and monitored closely with a monitor (IE-12 VET, TOOTOO MEDITECH, China). After the chinchilla lost its righting reflex, the isoflurane concentration was adjusted to 2.5% for maintenance anesthesia. During the operation, blood oxygen clips were used to monitor blood oxygen saturation and 3-lead electrodes were used to monitor heart rate. The surgical area was shaved and disinfected alternately with iodophor and alcohol. The medial access was selected for surgery, and the subcutaneous tissue and muscle were gradually separated. After exposing the broken end of the fracture, the surrounding callus was carefully cleaned, and the broken end was slightly polished with a bone tumbler. The diameter of the 25-gauge needle is 0.5 mm, which can be inserted into the radial medullary cavity, so the 25-gauge needle is used as the IM pin for fixation. After the radius was identified, the needle tip was directed toward the distal radius. We first inserted the needle retrogradely into the proximal radial medullary cavity, but to prevent the needle from being over-inserted and unable to be removed, and then inserted it downward into the distal medullary cavity. After the needle is inserted, the anastomosis of the fracture end is confirmed, and then the muscle and skin are sutured with 5-0 PGA. X-rays were taken immediately after surgery (Fig. 2A). X-ray results showed that most of the callus at the broken ends of radius and ulna had been removed. The radius and ulna were in a straight line, but there was no complete connection between the broken ends. It is recommended that the owner review the X-rays after one month.

### Outcome and follow-up

Postoperative management included meloxicam (0.3 mg/kg, PO, Melocam, Hanvet, China) for 3 days, enrofloxacin (10 mg/kg, SC, BAYTRIL, BAYER, Germany) for 3 days, and wound disinfection 3 times a day. After the surgery, the chinchilla was put on an Elizabethan ring and confined to a small space to reduce movement. Adequate amounts of vitamins and calcium should be added to their daily diet to strengthen their bones. Ten days after surgery, the wound was growing well, and the skin sutures were removed. The limb was able to support weight, and pain tests were normal, but grasping reflex was not showing. At a follow-up visit 24 days after the operation, the grasping posture was occasionally seen, and the X-ray of the forearm was again used, which showed a slight rotation of the needle (Fig. 2B). Due to the impact of COVID-19, the pet owner could not take the chinchilla to the hospital for follow-up visits, so follow-up visits were made by video recording and video chatting. Thirty one days after the operation, it was observed that the chinchilla could grasp the timothy grass, support the ground, assist in jumping, etc. In contrast, previously it had been in a suspended position and could not fully perform the above actions. At this time, the affected limb of the chinchilla regained the ability to grasp and grab food.

## Discussion

Chinchilla fractures can occur for many reasons, some of which include inadequate diet, an inappropriate cage design, hazardous cage components, and poor management. To maintain chinchillas’ strong bones, veterinarians need to make sure owners provide them with a healthy balanced diet. When a chinchilla jumps into the cage, its tiny legs frequently land between the ramp and the grid spacing at the bottom. Additionally, the chinchilla’s legs could become entangled in a metal grid cage with a wide bottom spacing. Considering this, it is preferable to switch to a cage with a solid bottom and a very small grid spacing. Additionally, when playing outside of the cage, owners should pay closer attention to how it moves to avoid tripping and unintentional stepping. Once a fracture occurs, it is recommended to seek medical attention as soon as possible. During the moving process, pay attention to laying shredded paper or loose sheets on the bottom of the pet cage, and then gently put the chinchilla in it and cover it with a sheet or other items to protect the chinchilla from stress factors such as extreme temperature or noise.

A chinchilla with a fracture is very painful at the beginning, unable to move, and the fracture site is swollen. In general, conservative treatment, including pain relief and reduced activity, is recommended for fractures that have malunion and most of the function is preserved. However, considering that chinchillas often fell during jumping and climbing and had pain in the right forelimb, many pet owners wanted further surgical reduction. Bone rubbing may be heard when manipulating the fractured area due to friction between the fractured ends. Internal fixation of fractures in small exotic mammals is challenging (Miwa and Calvo Carrasco 2019). They are smaller than dogs and cats, which means they have less room to operate and are more susceptible to damage tissue and bones than dogs and cats. When bone fractures occur in chinchillas, it is necessary to find materials of suitable size and to choose the treatment method flexibly according to the fracture site. Small rodents have low bone mass, thin cortical bone (Miwa and Calvo Carrasco 2019), and little covering of soft tissue, making it difficult for bone plates to function (Dan 2012). Improper internal fixation materials may lead to material loosening in the later stage, leading to infection and necrosis of the affected limb. Complications of surgical fixation are common, including internal fixation loosening and infection, wound non-healing, distal ischemic necrosis, and even self-mutilation. The movement of fracture ends can lead to delayed union and nonunion of fractures (Capello 2006). If surgical fracture stabilization fails or is not indicated, hindlimb amputation should be considered, and chinchillas usually adapt well after amputation (Mitchell and Tully 2008). Therefore, when a chinchilla suffers a fracture, the owner should be informed of the worst prognosis.

Chinchillas typically need fewer than 1 mm IM pins to fixate fractures of the radius and ulna. The radial medullary cavity of this chinchilla measured 0.7 mm in diameter. Nevertheless, a 25-gauge needle was used in its place because our hospital did not have an IM pin of this size. We believe that this needle provides adequate reduction and bending resistance like IM pin, but does not counteract rotational and shear stresses (Miwa and Calvo Carrasco 2019). The main function of the forelimbs of chinchillas is not a movement, so a slight rotation of the stump is acceptable. With simply a pin, it can be accomplished in several small mammals (Sánchez-Migallón Guzmán and Kapatkin 2021). Although needle rotation was indeed encountered in this case, no other problems were found in the follow-up, such as infection necrosis, fracture nonunion, etc. Therefore, we consider this fixation to be reliable.

The forelimbs of chinchillas do not have excessive muscles and the skin is tight, so care must be taken during the operation to reduce or avoid damage to muscles, blood vessels, and nerves. Ideally, the internal fixation pin should fill about 70% of the diameter of the medullary canal (Miwa and Calvo Carrasco 2019). In a study in rabbits, needle filling up to 75% of the medullary cavity diameter resulted in iatrogenic fractures (Sánchez-Migallón Guzmán and Kapatkin 2021). But at present, chinchillas have no research reports in this area.

According to statistics, among the 19 chinchillas involving long bone fractures, seven had tibial fractures, five had fractures of the radius and ulna, and four had fractures of the phalanges. Of all fractured chinchillas, six were amputated, five underwent splinting, and three underwent intramedullary fixation (Desprez et al. 2016). From the above data, we can preliminarily clarify the types of common extremity fractures in chinchillas and their treatment methods. In addition, some specific treatments have been reported. For example, the repair of tibial fractures in chinchillas using only external fixators has been reported (Capello 2011b). It has also been reported that intramedullary fixation combined with external skeletal fixation was used to treat multiple metatarsal fractures of the chinchilla hindlimb, and the fractures healed completely 56 days after the operation (Desprez et al. 2016). Installation of external fixation brackets is a common surgical technique in small mammals (Sánchez-Migallón Guzmán and Kapatkin 2021). Fractures in guinea pigs are also common, and surgical treatment of fractures has also been reported. Fractures of the tibia and femur in guinea pigs have been treated with internal fixation with good results (Macedo et al. 2015; Pertiwi 2018; Sezer 2020). The use of 22G indwelling needles for fixation hamster tibia has also been reported (Capello 2011a). In this case report, unlike the reported operations, we are dealing with an old fracture, which means more difficult and challenging operation. To repair the fracture, the old fracture needs to be destroied and then fixed. Moreover, the fixing material we used is not the often-mentioned IM pin, but a disposable injection needle. To the best of our knowledge, this is the first report of the use of disposable syringe needle for the treatment of old radial and ulnar fractures in chinchillas.

Chinchillas are not easy to manage after a fracture, either in the hind limbs or the forelimbs. Therefore, the prognosis is closely related to postoperative management. After surgery, an Elizabethan circle is required. In addition, it is usually recommended that the owner put the chinchilla in a cage with less space to reduce jumping. Vitamin C and calcium should be supplemented in the daily diet to help fractures heal. If the surgical fit is good, a stable callus can form in 7-10 days (Dan 2012).

## Conclusions

In conclusion, in this case, we describe diagnosis, treatment and prognosis of an old fracture of radius and ulna in a chinchilla. The use of syringe needles achieved good results, which provides a reference for the diagnosis and treatment of such diseases.

## Data Availability

Not applicable.

## Abbreviations

IM pin: Intramedullary Pin
